# Office Paper Platform for Bioelectrochromic Detection of Electrochemically Active Bacteria using Tungsten Trioxide Nanoprobes

**DOI:** 10.1038/srep09910

**Published:** 2015-04-20

**Authors:** A. C. Marques, L. Santos, M. N. Costa, J. M. Dantas, P. Duarte, A. Gonçalves, R. Martins, C. A. Salgueiro, E. Fortunato

**Affiliations:** 1 Departamento de Ciência dos Materiais, CENIMAT|I3N and CEMOP/UNINOVA, Faculdade de Ciências e Tecnologia – Universidade Nova de Lisboa, 2829-516 Caparica, Portugal; 2 Departamento de Ciências da Vida, CIGMH, UCIBIO, Faculdade de Ciências e Tecnologia – Universidade Nova de Lisboa, 2829-516 Caparica, Portugal; 3 Departamento de Química, UCIBIO-REQUIMTE, Faculdade de Ciências e Tecnologia - Universidade Nova de Lisboa, 2829-516 Caparica, Portugal

## Abstract

Electrochemically active bacteria (EAB) have the capability to transfer electrons to cell exterior, a feature that is currently explored for important applications in bioremediation and biotechnology fields. However, the number of isolated and characterized EAB species is still very limited regarding their abundance in nature. Colorimetric detection has emerged recently as an attractive mean for fast identification and characterization of analytes based on the use of electrochromic materials. In this work, WO_3_ nanoparticles were synthesized by microwave assisted hydrothermal synthesis and used to impregnate non-treated regular office paper substrates. This allowed the production of a paper-based colorimetric sensor able to detect EAB in a simple, rapid, reliable, inexpensive and eco-friendly method. The developed platform was then tested with *Geobacter sulfurreducens*, as a proof of concept. *G. sulfurreducens* cells were detected at latent phase with an RGB ratio of 1.10 ± 0.04, and a response time of two hours.

Electrochemically active bacteria (EAB) transfer electrons outside their cells toward insoluble electron acceptors during their respiration^1^,^2^. These organisms can be found in diverse environments, such as oceans, lakes and river sediments as well as domestic and industrial wastewater streams^3^. The most common application of EAB is the production of electrical current from the bacterial metabolism in microbial fuel cells (MFCs)^1^,^2^. Nevertheless, significant optimization of MFCs is still required. Besides electricity production, other applications of EAB include wastewater treatment, hydrogen production and *in situ* monitoring of microbial respiration^4^,^5^. Further research into the physiology and ecology of EAB is essential to design microorganisms with improved electron transfer capabilities^6^,^7^. Nowadays, the available screening methods are based on MFC principles, such as, voltage-based screening assay^8^, microfabricated MFC array^4^ and U-tube-shaped MFC^9^. However, these methods are relatively slow (~ 5 to 6 days) and expensive. The development of rapid and simple screening methods using low cost and available materials is today a key issue to identify EAB. The use of electrochromic materials has emerged recently as an attractive mean for colorimetric identification and characterization of EAB, making use of their reducing ability. Yuan *et al*.^10^ have described an optical probe method for the detection of EAB in solution using an electrochromic (EC) material, tungsten trioxide (WO_3_). EC materials change their optical properties (between two coloration states) with the appliance of a sufficient electrochemical potential. In this work, the bioelectrochromic response is achieved by an electron-transfer (redox) process of the EAB^11^,^12^. The electrochemical reaction that results in the chromic response of WO_3_ can be described considering the injection of an equimolar amount (*x*) of positive ions (M^+^) and electrons (e^−^) as depicted in Equation 1:
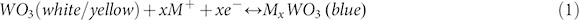
Typical cations are H^+^, Li^+^ and Na^+^, with a stoichiometry that can vary between zero and one^13^. In this work, nanostructures of WO_3_ were synthesized through a microwave assisted hydrothermal synthesis, which offers significant advantages, such as control over the crystal growth, shape and size, low number of impurities, improved product yield, low processing temperature, high homogeneity, very rapid heating to treatment temperature, low cost and easy synthesis^14^,^15^,^16^,^17^,^18^. The WO_3_ nanoparticles were integrated in a wax-printed office paper platform as an active layer for EAB detection. The fabrication of the paper-based sensor comprises two main steps: (i) wax printing of the hydrophobic patterns on the office paper and (ii) drop casting of the WO_3_ nanoparticles dispersion. Wax printing is a micropatterning method proposed by G. M. Whitesides' group for paper-based microfluidics^19^. Initially, a wax printer deposits a layer of wax onto the paper's surface that is then fused to penetrate throughout the paper thickness rendering it hydrophobic^20^,^21^,^22^,^23^,^24^. This method represents nowadays the fastest and simplest paper patterning process, allowing the formation of well-defined, sub millimeter-sized hydrophilic wells surrounded by hydrophobic barriers^19^,^22^. The proposed paper-based sensor was successfully used to test the presence of electrochemically active *Geobacter sulfurreducens*^25^ cells. The presence of these cells in the sample induces tungsten bronze formation displaying a deep blue color that highly contrasts with the white background provided by the paper platform. The color discrepancy between a positive (deep blue) and negative (white) result can be easily scrutinized by visual comparison or RGB analysis performed onto a digital photograph. Here, is reported for the first time the use of a low cost paper-based sensor to optically detect the presence of a well-known and deeply studied EAB, *Geobacter sulfurreducens*^26^,^27^,^28^.

## Results and discussion

### Tungsten trioxide synthesis

**Figure 1:**
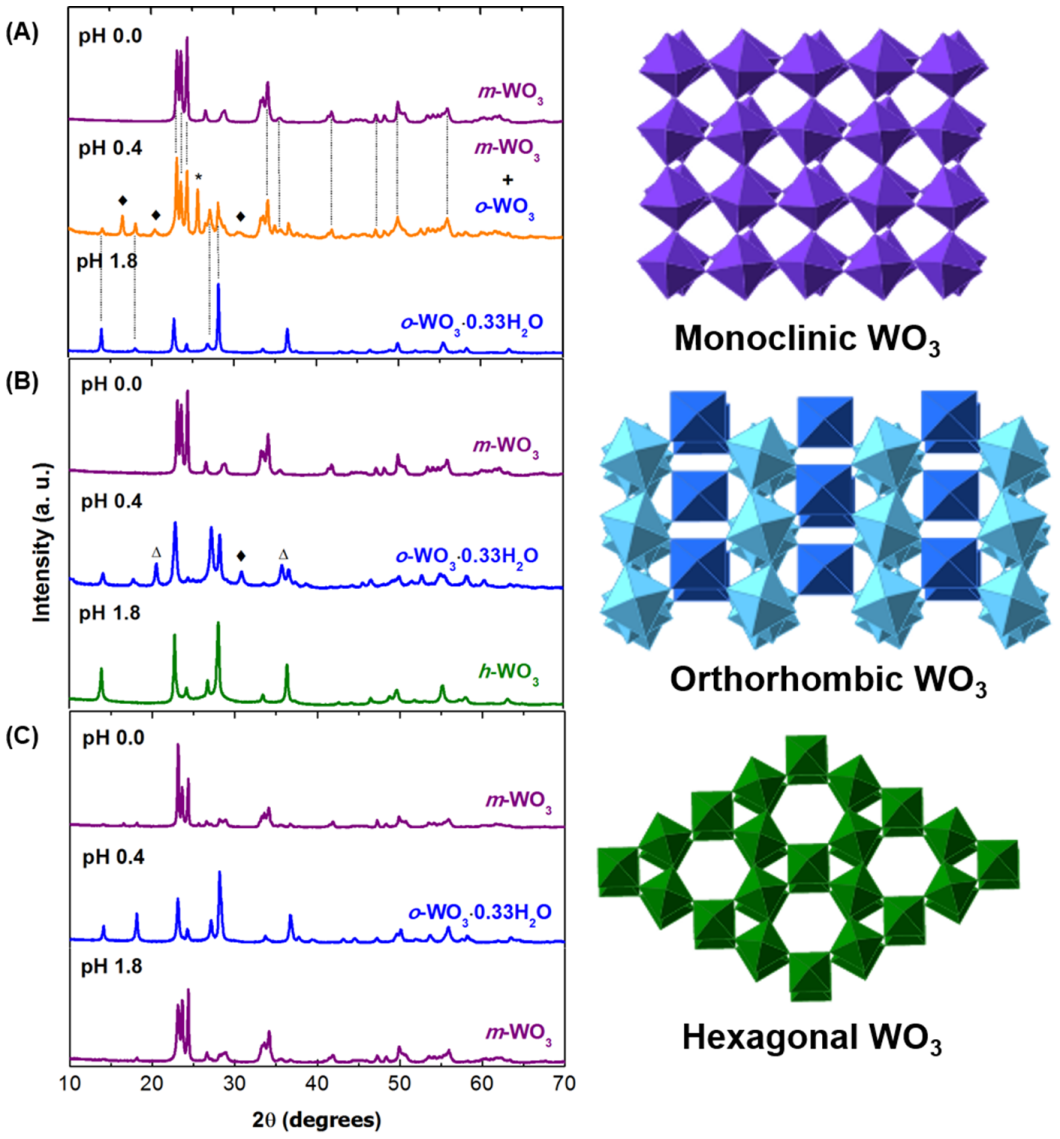
XRD diffractograms of the WO_3_ nanoparticles. (A) WO_3_ nanoparticles synthesized from Na_2_WO_4_·2H_2_O, NaCl solutions; (B) WO_3_ nanoparticles synthesized from Na_2_WO_4_·2H_2_O, Na_2_SO_4_ solutions; (C) WO_3_ nanoparticles synthesized from PTA solutions. The peaks marked as * and ♦ are characteristic of Na_2_WO_4_·2H_2_O and H_2_WO_4_ structures. The peaks marked as Δ are non-identified. The crystalline structures were produced with the CrystalMaker software (Centre for Innovation & Enterprise, Oxford).

The crystallographic structure of the synthesized WO_3_ nanoparticles was determined by X-ray diffraction (XRD) (Figure 1) and corroborated by Fourier transform infrared spectroscopy (FT-IR) (Fig. S1). Tungsten oxides follow a well-known ReO_3_-type structure built up of layers containing distorted corner-shared WO_6_ octahedra. The growing process of WO_3_ nanostructures can be described in three major steps: (i) formation of the tungstic acid (H_2_WO_4_), (ii) formation of WO_3_ clusters by decomposition of H_2_WO_4_ and (iii) growth of WO_3_ crystal nucleus^29^. In the synthesis with sodium tungstate dihydrate (Na_2_WO_4_·2H_2_O) as precursor and NaCl as structure-directing agent (SDA) (Figure 1A), WO_3_ nanoparticles grow in a monoclinic (*m*-WO_3_) crystallographic structure (ICDD #00-043-1035) at pH 0.0 and orthorhombic (*o*-WO_3_·0.33H_2_O) (ICDD #01-072-0199) at pH 1.8. At pH 0.4 the WO_3_ nanoparticles are a mixture of the two phases, monoclinic and orthorhombic, together with the precursor (marked with *) and tungstic acid (marked with ♦). Using Na_2_SO_4_ as SDA (Figure 1B), orthorhombic and hexagonal (*h*-WO_3_) (ICDD #01-075-2187) phases were obtained at pH 0.4 and pH 1.8, respectively. At pH 0.4 the sample also shows a peak assigned to the acid tungstic (♦) and two unidentified peaks (Δ) that are due to lattice distortions of the crystallographic structure, as previously reported for WO_3_ nanoparticles prepared by hydrothermal synthesis^30^. Finally, using peroxopolytungstic acid (PTA) as precursor (Figure 1C), the crystallographic structure of the synthesized nanopowder is monoclinic for the lowest and higher pH values, which is in agreement with previous reports, although with different crystallographic plane intensities^29^. For the intermediate pH value, the WO_3_ nanoparticles present an orthorhombic phase. The FT-IR analysis is in accordance with the crystallographic structures attributed by XRD. However, the samples prepared with PTA precursor also revealed the presence of a W = O vibration bond that are assigned to some impurities. In general, the formation of nanoparticles is favourable for pH values lower that 2.0, however at pH 0.4 tend to form bundle structures and a mixture of phases and/or impurities^31^.

**Figure 2:**
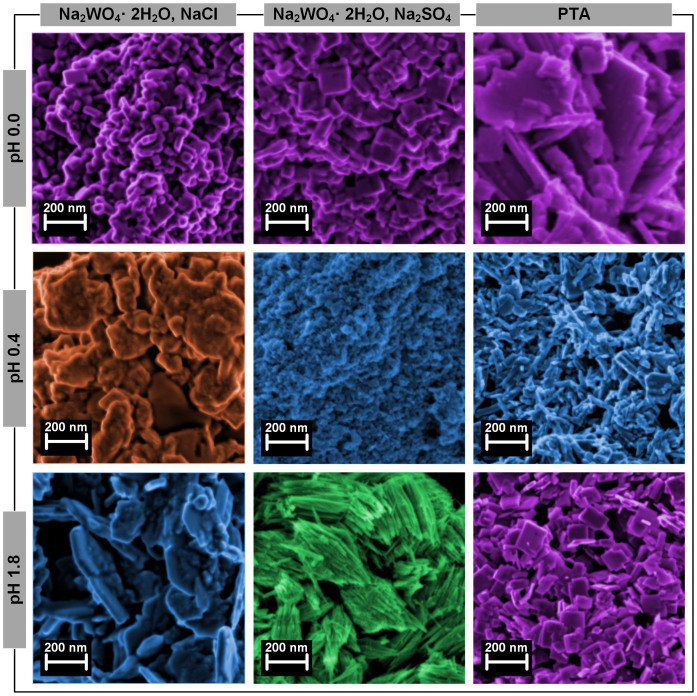
SEM images of the synthesized WO_3_ nanoparticles. The images are false colored (GIMP software) for better understanding. The different colors are related with the XRD diffractograms for each crystalline structure.

Figure 2 shows the morphological analysis performed by scanning electron microscopy (SEM). The WO_3_ nanoparticles synthesized with Na_2_WO_4_·2H_2_O show different morphologies: (i) nanocubes assigned to *m*-WO_3_, (ii) nanosheets assigned to *o*-WO_3_·0.33H_2_O, (iii) nanowires assigned to *h*-WO_3_ and (iv) bundle structures. Regarding the WO_3_ nanoparticles synthesized with PTA, the obtained structures present well-defined edges with nanosheet-like morphology at pH 0.0, a mixture of nanowires and nanocubes at pH 0.4 and single nanocubes at pH 1.8.

The sulphate ions added to the synthesis process, act as capping agents covering some facets of WO_3_ crystal nuclei. At pH 1.8 a faster growth rate along *c*-axis is observed, yielding to one-dimensional wire/rod-like structures. In the meantime, a certain amount of sodium cations is required as stabilization ions for the hexagonal and triangular tunnels in the formation of *h-*WO_3_^15^,^31^,^32^,^33^. When chloride ions are added it is believed that a similar process occurs prompting the growth of the nanoparticles in a specific direction^34^.

Additionally, electrochemical impedance spectroscopy (EIS) was performed in all the samples, in order to compare the conductivity of the nanostructures, since this affects its electron transfer ability during the electrochromic process. The Bode plots represented in **Fig. S2** display, in general, lower impedances values for the orthorhombic and hexagonal crystallographic structures, which is in accordance with the literature^35^,^36^,^37^. The results obtained for hexagonal crystallographic structure (Table S1) are explained by its high surface area and tunnel structure.

### Office paper as a platform for EAB identification

**Figure 3:**
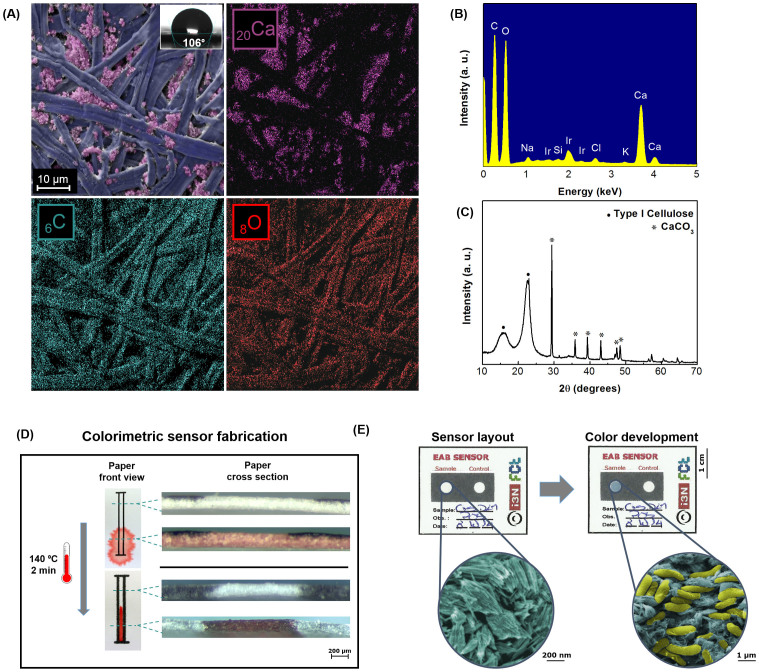
Office paper characterization. (A) SEM image and EDS map; (B) EDS spectrum; (C) XRD diffractogram; (D) Hydrophobic barriers formation; (E) Photograph of a positive result in the developed paper-based sensor with WO_3_ nanoprobes for the colorimetric detection of EAB (*Geobacter sulfurreducens* cells in yellow and hexagonal WO_3_ nanoparticles in blue). The images are false-colored (GIMP software) for better understanding of the different materials.

The above described WO_3_ nanoparticles were used as an active layer in a regular office paper substrate in order to develop a colorimetric electrochemical device for EAB detection. This paper is optimized for printing, and therefore has a more uniform surface, lower porosity and higher hydrophobicity (water-contact angle of 106°) when compared to chromatography paper (water-contact angle of 12°)^22^, the most common type employed in paper-based devices. Office paper allows a superficial adhesion of the WO_3_, which facilitates the interaction of EAB with the electrochromic nanoparticles, promoting an intense and uniform coloration of the test well. SEM-EDS and XRD analysis (Figure 3A–C) revealed a high-density structure of intertwined cellulose fibers with a cylindrical and flat shape, and the presence of agglomerates, especially calcium carbonate, as confirmed by a FT-IR analysis (Fig. S3).

**Figure 4:**
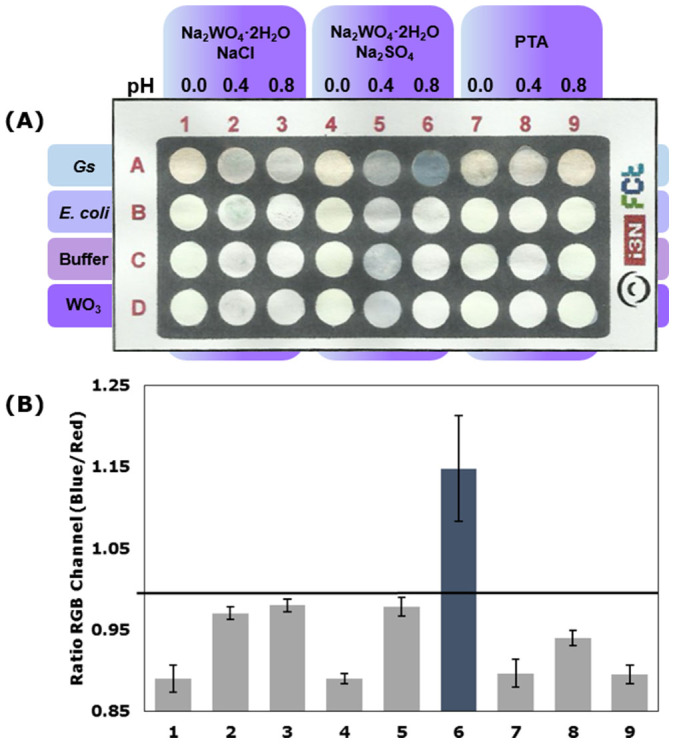
EAB detection. (A) Paper-based sensor photograph of the Colorimetric assays of all synthesized WO_3_ nanoparticles at 5 g/L; (B) RGB analyses for all the samples in contact with *Geobacter sulfurreducens* (*Gs*) cells. (Results recorded after 4 hours) The horizontal line represents the threshold of 1 considered for discrimination between positive and negative and the results represent the average of three independent measurements with the respective error bars indicative of the standard deviation.

The sensor layout was designed to resemble a conventional microplate (Figure 4A) for parallel assays and prototyped into single-use sensors containing only a test and a control wells (Figure 3D–E). The layout was then patterned by wax printing, a method previously optimized by M. N. Costa *et al*.^22^, that increases the surface hydrophobicity (water-contact angle of 119°) ensuring no cross contamination between adjacent samples as well as the confinement of the WO_3_ nanoparticles dispersion to one particular area. A thermal analysis of the office paper (Fig. S4) was also performed in order to guarantee that the material could withstand the heating process. The patterned office paper was then impregnated with the synthetized WO_3_ nanoparticles by a drop casting process. A drop was spotted on each well delimited by the hydrophobic pattern, in order to create the electrochromic layer.

### Colorimetric assays

A screening colorimetric assay of all the synthesized WO_3_ nanoparticles was performed in the developed paper-based sensor (Figure 4A) and in the conventional 96-well plate (Fig. S5). For the paper platform, an RGB analysis of the results was carried out using ImageJ software, and the ratio of the average intensities in blue and red channels was recorded (Figure 4B). An electrochromic response translated by the deep blue color of the tungsten bronze (Equation 1) was achieved with sample 6, which corresponds to the synthesized hexagonal WO_3_ nanowires. The *h*-WO_3_ nanoparticles has attracted much attention due to its well-known tunnel structure where openness degree is higher when compared to the layered structure of orthorhombic or monoclinic geometries. This feature results in an easier intercalation of cations to form tungsten bronzes and concomitant enhancement of the electrochromic properties^14^,^38^. Moreover, the one-dimensional nanowire shape originates a structure with a high surface area and increased surface atom density that can easily interact with the EAB. Additionally, the electrochromic response was also observed in the conventional assay with other crystallographic WO_3_ structures, due to the higher concentration of the nanoparticles and enhanced interaction with EAB (Fig. S5).

**Figure 5:**
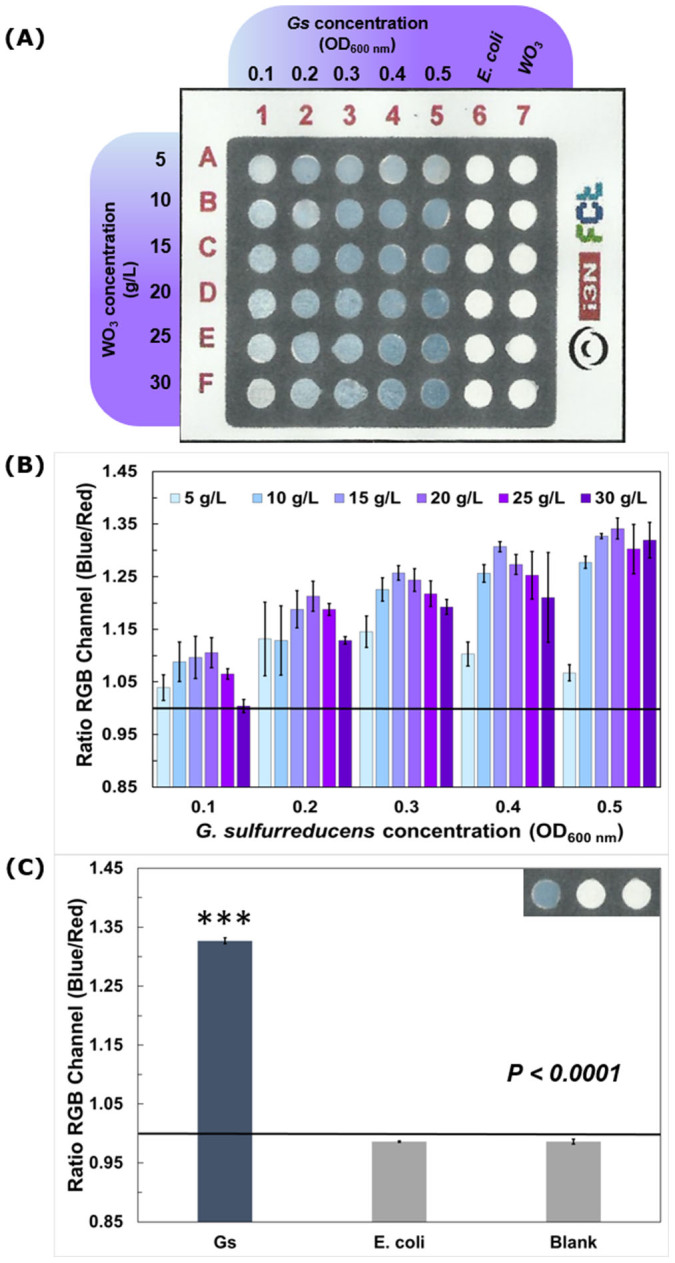
EAB detection. (A) Paper-based sensor photograph of the Colorimetric assays of *h*-WO_3_ nanoparticles at different concentrations; (B) RGB analyses of *h*-WO_3_ nanoparticles at 15 g/L in contact with *G. sulfurreducens* cells, with the negative control *E. coli* and a blank test. (Results recorded after 2 hours) The horizontal line represents the threshold of 1 considered for discrimination between positive and negative and the results represent the average of three independent measurements with the respective error bars indicative of the standard deviation.

For the fabricated paper-based sensor, the bioelectrochromic response is conditioned by the time that the cell suspension drops (V = 50 µL) take to dry (approximately 4 hours). Therefore, an equal scale down on the well's diameter (d = 3.38 mm) and in the sample volume (V = 20 µL) was carried out, with a reduction (2.5 times smaller) from the first paper-based sensor (Figure 4A). Additionally, an optimization assay was carried out (Figure 5A) to evaluate the influence of the *h*-WO_3_ nanoparticles concentration in the detection of EAB. Figure 5B represents the RGB analysis of the resulting color of the *Geobacter sulfurreducens* cells in contact with *h*-WO_3_ nanoparticles. From this analysis it is possible to conclude that 15 g/L and 20 g/L *h*-WO_3_ nanoparticles dispersion renders higher RGB ratios when compared to the other studied concentrations. Moreover, 15 g/L nanoparticles dispersion presents a linear response to the increasing *G. sulfurreducens* cells concentration. Therefore, henceforward the sensors were produced with *h*-WO_3_ nanoparticles dispersion at 15 g/L, with the same ratio nanoparticles per area than the first sensor (0.014 g/mm^2^). With the described scale-down the response time decreased to half the time (approximately 2 hours).

Figure 5C represents an RGB analysis of a positive control (*Geobacter sulfurreducens*), a negative control (*Escherichia coli*) and a blank well (no sample) for background information. *Geobacter sulfurreducens* cells in a late-exponential phase of growth (Abs_600 nm_ ~ 0.5) display an RGB ratio of 1.33 ± 0.005, while the negative control, *Escherichia* *coli* under the same conditions, and blank sample display an equal ratio of 0.99 ± 0.001/0.004. This result reveals a clear statistically significant difference between a positive and negative sample (P < 0.0001), proving the specificity of the developed paper-based device (Table S2). Moreover, it was also possible to detect EAB at latent phase (Abs_600 nm_ ~ 0.1), with an RGB ratio of 1.10 ± 0.040, thus confirming that the sensor here described is sensitive for low concentrations of EAB.

## Conclusions

Electrochemically active bacteria are ubiquitous in nature and have the ability to transfer electrons outside their cells, a feature that can be applicable in electricity production, which is of the outmost importance in an energy dependent world.

However, the number of identified species is still very limited and their electron transfer mechanisms are feebly understood, caressing of feasible techniques to allow the detection of these bacteria as well as to facilitate the study of their physiology and electron transfer mechanisms.

This work reports a paper-based sensor with WO_3_ nanoprobes to optically detect the presence of these bacteria.

In order to study and optimize the electrochromic response to EAB cells, three different WO_3_ crystallographic structures (monoclinic, orthorhombic and hexagonal) were synthetized with the use of different precursors (Na_2_WO_4_·2H_2_O and PTA) and structure-directing agents (NaCl and Na_2_SO_4_) as well as by varying solution pH.

The performance of the produced structures as electrochromic material for EAB detection was evaluated in a screening colorimetric assay both in the conventional method, using a 96-well microplate, and in an office paper platform, with the same dimensions and produced through wax-printing method. Both assays achieved a successful detection of EAB *Geobacter sulfurreducens* with a high color contrast for the *h*-WO_3_ nanowires. This enhanced bioelectrochromic response is in accordance with mentioned above that this structure presents an increased surface area and surface atom density proving to be the ideal material for EAB electrochromic detection.

The conventional assay performed in a solution based reaction also demonstrated a mild colorimetric response with other WO_3_ structures, due to the easier contact with *G. sulfurreducens* cells.

Aiming to reduce the response time, sample and reagents volume necessary for the test, a scale down paper-based device was tested with a 2.5 × reduction of wells diameter. The mentioned scale down allowed a reduction in the response time for 4 hours to 2 hours.

The described paper-based sensor showed a RGB ratio above 1 for a sample of *Geobacter sulfurreducens* cells at latent phase, thus providing a reliable, inexpensive, eco-friendly and simple approach to identify EAB. Moreover, this method can also be used in screening assays to aid in the understandings of the influence of certain proteins in the electron transfer chain, thus allowing further refining in the performance of the mentioned applications.

### Experimental section

#### Tungsten trioxide synthesis

Several types of nanoparticles were synthesized through a microwave assisted hydrothermal method. Two different precursors were used (0.8 g): sodium tungstate dehydrated (Na_2_WO_4_·2H_2_O), (Fluka, 99%) and peroxopolytungstic acid (PTA), prepared by oxidation of tungsten (W) with hydrogen peroxide (H_2_O_2_), previously described^39^,^40^.

Two different salts were used as structure directing agents (SDA): NaCl (0.3 g) (Panreac, 99.5%) and Na_2_SO_4_ (0.72 g) (Panreac, 99%) with the Na_2_WO_4_·2H_2_O precursor. For each set of precursor, 3 M hydrochloric acid (Sigma-Aldrich, 37% by weight) was added to the solutions thus forming the acid tungstic (H_2_WO_4_) intermediate, and the final pH values were set as 0.0, 0.4 and 1.8. In the samples produced from PTA precursor the influence of SDA was not studied.

The solutions were then transferred into a 35 mL vessel, sealed, and heated in a microwave reactor (Discover SP, CEM Corporation, Matthews, NC, USA) at a constant power of 100 W for 1 h. All nine nanopowders were collected by centrifugation (4000 rpm, ~ 30 min), and dried (80°C, ~ 12 h). Solids were then grounded in a mortar to obtain fine powders and stored for further characterization.

#### Paper sensor fabrication

A4 (210 × 297 mm) sheets of an office paper (300%, Portucel Soporcel, Setúbal, Portugal) were fed to the manual feed tray of a commercial solid ink printer (Xerox ColorQube 8570, Xerox Corporation, Norwalk, CT, USA) designed to print a wax-based ink. All the prototypes used in this work were designed in Adobe Illustrator (Adobe Systems Software, Ireland). The prototypes were projected to resemble 96 and 384-well plates with d = 0.7 and 0.38 cm, respectively.

The printed patterns were placed on a hot plate (Heidolph MR HeiTec, Schwabach, Germany) at 140°C for 2 min, allowing the wax to melt and spread vertically through the whole thickness of the paper, creating the desired hydrophobic pattern. Each well was functionalized with 40 µL and 10 µL of WO_3_ nanoparticles dispersion for the sensor with d = 0.7 cm and d = 0.38 cm, respectively. The papers were allowed to dry at room temperature overnight. The final paper sensors were stored at room temperature in a dark box with no light interferences.

#### Bacterial growth

*Geobacter sulfurreducens* (strain ATCC 51573/DSM 12127/PCA) was inoculated under strict anaerobic conditions in NBAF medium with the following composition: 0.04 g/L CaCl_2_·2H_2_O, 0.1 g/L MgSO_4_·7H_2_O, 1.8 g/L NaHCO_3_, 0.5 g/L Na_2_CO_3_·H_2_O, 0.19 mg/L Na_2_SeO_4_, DL vitamins, 100X NB salts and NB mineral elixir, with 10 mM sodium acetate as the electron donor and 40 mM sodium fumarate as the electron acceptor. To remove oxygen, the NBAF medium was placed in 10 mL pressure tubes and gassed with an 8:2 mix of N_2_:CO_2_. After the NBAF medium deaeration and sterilization, 2% of yeast extract, 1% of 100 mM pH 7.0 cysteine and 1% of *Geobacter sulfurreducens* cells from a frozen stock were added to the culture medium under an anaerobic atmosphere. Cultures were grown at 30°C. The bacterial growth was followed through absorbance readings at 600 nm (Fig. S6). *Escherichia coli* (strain BL21 (DE3)), used as a negative control on the colorimetric assays, was inoculated in LB medium (10 g/L tryptone, 5 g/L yeast extract, and 10 g/L NaCl) and incubated at 37°C, 200 rpm.

#### EAB-WO_3_ colorimetric assay

An anaerobic chamber (LABstar Glove Box Workstation, MBRAUN, Garching, Germany, O_2_ < 0.1 ppm) was used to perform all the colorimetric assays. *Geobacter sulfurreducens*, grown until late-exponential phase, and *Escherichia coli*, in the same conditions, were collected by centrifugation at 6000 rpm for 5 min and then suspended in a buffer (30 mM Na_2_HPO_4_·2H_2_O, 30 mM KCl and 30 mM CH_3_COONa, pH 6.53). Cell cultures (50 µL and 20 µL for the sensor with d = 0.7 cm and d = 0.38 cm, respectively) were added to the respectively well and the drops were allowed to dry at room temperature.

#### Data acquisition and analysis

The colorimetric paper-based results were recorded with a digital scanner (All-in-One Printer 1050A HP, Hewlett-Packard Development Company, L.P., Palo Alto, CA, USA) with a 300 dpi resolution. Color development analysis was performed with ImageJ software (National Institutes of Health, Bethesda, Maryland, USA) through an RGB control. A one-way ANOVA analysis, with Tukey's multiple comparison test, using GraphPad (San Diego, CA) was used to validate the results. The one-way analysis of variance was used to test for differences between two groups of data (positive and negative results). Additional statistical analysis of the differences was carried out using Tukey's multiple comparison procedures.

#### Characterization

The crystallographic structure of the WO_3_ nanoparticles and the office paper were determined by XRD (X'Pert Pro, PANalytical, Almelo, Netherlands) with CuKα target and wavelength of 1.5406 Å. The nanoparticles' morphology and paper was characterized by SEM-EDS (Carl Zeiss AURIGA Crossbeam SEM-FIB, Oberkochen, Germany) and their chemical structure by FT-IR (Nicolet 6700 FT-IR, Thermo Electron Corporation, Waltham, MC, USA) with a diamond crystal attenuated total reflectance (ATR) accessory. The electrochemical impedance spectroscopy was performed in a potentiostat (600TM Gamry Instruments) with WO_3_ powders in a form of pellets made at a pressure of 8 tons, with a diameter of approximately 1 cm and a thickness of 1–3 mm. The electrochemical cell consisted in two stainless steel electrodes in each side of the pellet compacted in a homemade cell.

#### Scanning electron microscopy of biological samples

*Geobacter sulfurreducens* cells alone and in contact with WO_3_ nanoparticles were imaged using SEM. Before imaging, the mixture was fixed in 3% glutaraldehyde in a 0.2 M cacodylate buffer solution for 2 h, rinsed two times with 0.1 M cacodylate buffer solution, and dehydrated through graded ethanol (70, 95, and 100%, respectively, for 20 min each).

## Author Contributions

A.C.M., L.S., M.N.C., J.M.D., P.D. and A.G. contributed equally to the work and R.M., C.A.S. and E.F. supervised the work. All authors reviewed the manuscript.

## Supplementary Material

Supplementary Information Supplementary Information
