# Supplementary material for: Office Paper Platform for Bioelectrochromic Detection of Electrochemically Active Bacteria using Tungsten Trioxide Nanoprobes
Source: Sci Rep. 2015 Apr 20;5:9910. doi: 10.1038/srep09910 (PMC4402613; doi:10.1038/srep09910)
Supplement: Supplementary Information [file srep09910-s1.doc]

Supporting Information

**Office Paper Platform for Bioelectrochromic Detection of Electrochemically Active Bacteria using Tungsten Trioxide Nanoprobes**

A. C. Marques^1,3^, L. Santos^1^, M. N. Costa^1,2^, J. M. Dantas^3^, P. Duarte^1^, A. Gonçalves^1^, R. Martins^1^, C. A. Salgueiro^3,*^ and E. Fortunato^1,*^

1. **WO_3_ nanoparticles characterization**

Additional chemical and electrochemical characterizations were performed with the purpose of ample the tungsten trioxide synthesis study.

- 1. **Fourier transform infrared spectroscopy**

In order to confirm the bonds present in the synthesized WO_3_ nanoparticles an FT-IR analysis was performed (Figure S1). The assigned vibrations bands are in good agreement with the structures identified by XRD. Monoclinic and hexagonal structures show the typical bands of W‑O bonds whereas orthorhombic structures also show evidences of W=O and W‑OH terminal bonds, due to the water molecules in their structure. Note that the monoclinic WO_3_ nanoparticles from PTA solutions at pH 0.0 also show a band at ~1000 cm^-1^ corresponding to W=O bonds, that could be due to the remaining presence of the PTA precursor.


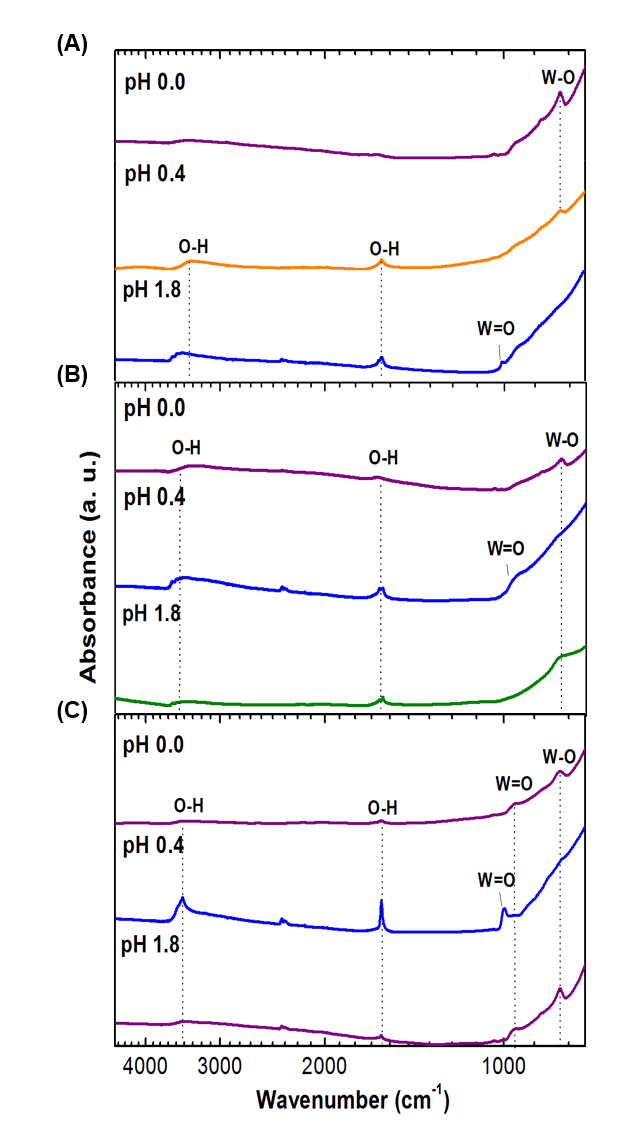


Figure S1 - FT-IR spectra of the WO_3_ nanoparticles: (A) WO_3_ nanoparticles synthesized from Na_2_WO_4_·2H_2_O, NaCl solutions; (B) WO_3_ nanoparticles synthesized from Na_2_WO_4_·2H_2_O, Na_2_SO_4_ solutions; (C) WO_3_ nanoparticles synthesized from PTA solutions. The colors of the spectra are related with the phase of the nanoparticles: purple – monoclinic phase; orange – mixture of monoclinic and orthorhombic phases; blue – orthorhombic phase and green – hexagonal phase.

- 1. **Electrochemical impedance spectroscopy**

Electrochemical impedance spectroscopy (EIS) was performed, in cylindrical pellets of pressed nanopowders, between two stainless steel electrodes, in the range of 1 and 10^6^ Hz.

From the impedance modulus (Bode plots) in Figure S2A, B and C, it is clear that the impedance is lower for the orthorhombic and hexagonal crystallographics, compared with the monoclinic, which is in accordance with the literature. The conductivity of the nanopowders was calculated from the fitting of the Nyquist plots, using the constant phase element (CPE) with diffusion model (inset Figure S2D) and using the equation (1):

σ = ɭ / (R × A) (1)

where ɭ and A are the thickness and the area of the cylindrical pellets, and R is the resistance obtained from the fitting of the Nyquist plot.

As an example, Figure S2D shows the Nyquist plot for the hexagonal crystallographic structure with the corresponding fitting curve. The good approximation to the experimental results shows that the chosen model was suitable for calculating the conductivity of the nanopowders (Table S1). Moreover, the low conductivity values of *h*-WO_3_ is in accordance with the tunnel structure and morphology of the nanoparticles, as described in the manuscript.

**
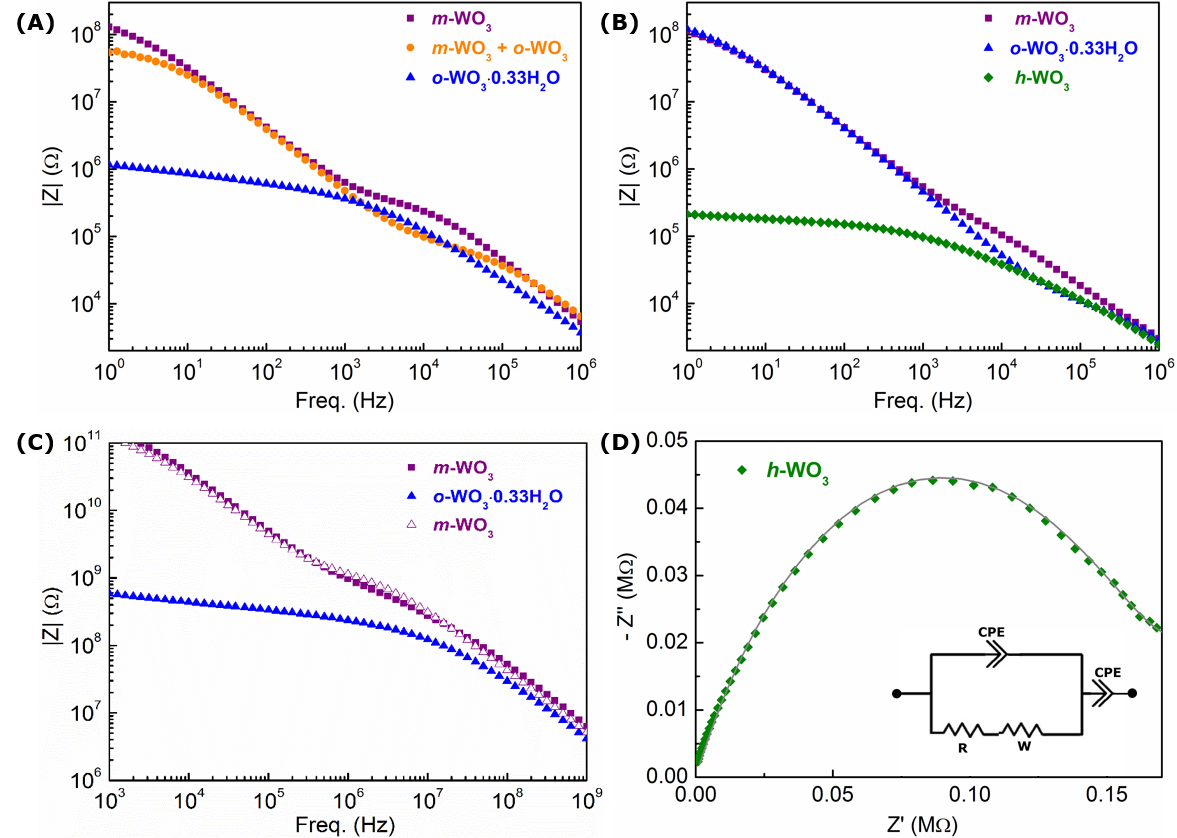
**

Figure S2 – Electrochemical characterization of the WO3 nanoparticles: Bode plots of WO_3_ nanoparticles synthesized from Na_2_WO_4_·2H_2_O, NaCl solutions (A); Na_2_WO_4_·2H_2_O, Na_2_SO_4_ solutions (B) and from PTA solutions (C). Nyquist plot of the hexagonal crystallographic structure obtained from the Na_2_WO_4_·2H_2_O, Na_2_SO_4_ at pH 1.8. The inset represents the impedance model used in the fittings (D).

Table S1 – Resistance and conductivity calculated values for all the samples.

| **Precursor** | **ADE** | **pH** | **Crystallographic**  **structure**  **P < 0.0001** | **Conductivity**  **(S.cm^-1^)** |
| --- | --- | --- | --- | --- |
| **Na_2_WO_4_·2H_2_O** | **NaCl** | **0.0** | *m*-WO_3_ | 2.49 × 10^-10^ |
|  |  | **0.4** | *m*-WO_3 +_ *o*-WO_3_ | 3.30 × 10^-10^ |
|  |  | **1.8** | *o*-WO_3_·0.33H_2_O | 4.71 × 10^-8^ |
|  | **Na_2_SO_4_** | **0.0** | *m*-WO_3_ | 2.60 × 10^-10^ |
|  |  | **0.4** | *o*-WO_3_·0.33H_2_O | 1.81 × 10^-10^ |
|  |  | **1.8** | *h-*WO_3_ | 2.41 × 10^-7^ |
| **PTA** |  | **0.0** | *m*-WO_3_ | 6.19 × 10^-11^ |
|  | **-** | **0.4** | *o*-WO_3_·0.33H_2_O | 1.66 × 10^-7^ |
|  |  | **1.8** | *m*-WO_3_ | 1.05 × 10^-10^ |

1. **Paper characterization**

The XRD diffractogram of the office paper (Figure 1) used as support for the colorimetric assays shows the characteristic peaks of type I cellulose (ICDD #00-056-1718/19) at 2θ = 15.88° and 22.6° that corresponds to miller indices (101) and (002), respectively. The other peaks can be assigned to CaCO_3_. This compound is commonly used as additive in the papermaking industry to improve the optical characteristics of the paper. The characteristic peaks of this component present on the paper are 29.47°, 39.49°, 47.52° and 48.62° with miller indices of (104), (113), (018) and (116), respectively. The CaCO_3_ identified (ICDD #01-072-1937) have a rhombohedral geometry with lattice constants of a = b = 4.99 Å and c = 17.08 Å and γ= 120°. An FT-IR analysis was also performed (Figure S2) to confirm the compounds present in the office paper, identified by XRD.

*
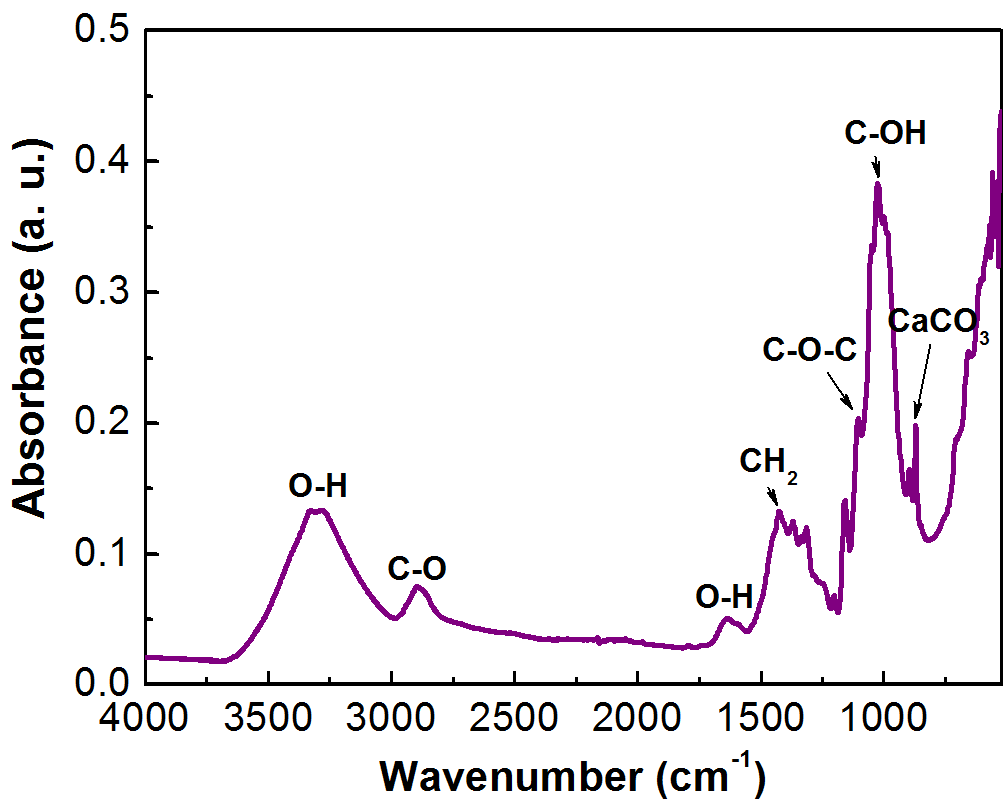
*

Figure S3 - FT-IR spectra of the office paper used as a platform on the colorimetric assays.

Additionally, a thermal analysis (Figure S3) was carried out, to assure that no transformations occur during the hydrophobic barriers formation at 140 °C. This analysis shows that only at temperatures above 300 °C it was detected 50% of mass loss associated to two endothermic peaks at 324.94 and 348.52 °C that correspond to the degradation of the cellulose fibres. For this reason, it was clear that no major mass losses occur until 140 °C.


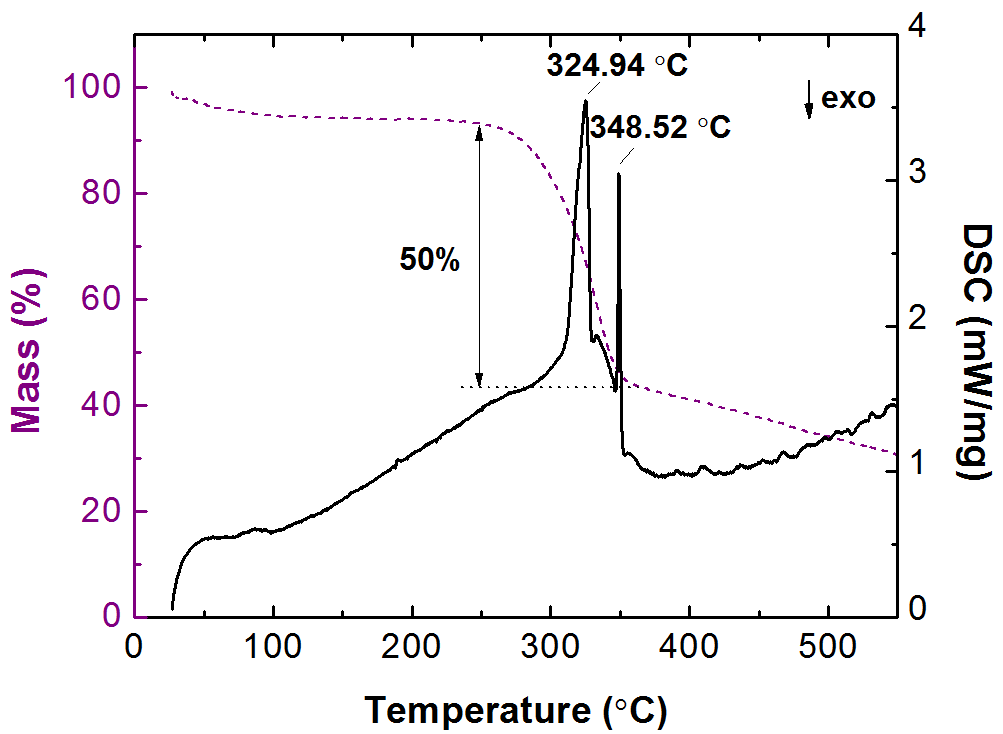


Figure S4 - Differential scanning calorimetry/thermo gravimetric (DSC/TG) analyses of the office paper.

1. **Colorimetric assays**

A conventional colorimetric assay on 96-well plate was performed (Figure S4). The image shows the results obtained for all the synthesized WO_3_ nanoparticles. As expected, the hexagonal WO_3_ nanoparticles has the better electrochromic response to EAB, translated by the deep blue color tungsten bronze. However, this deep blue color can also be observed in other WO_3_ nanoparticles samples, which shows that when the colorimetric assay is performed in a conventional way, monoclinic and/or orthorhombic structures can also provide a successful EAB detection.


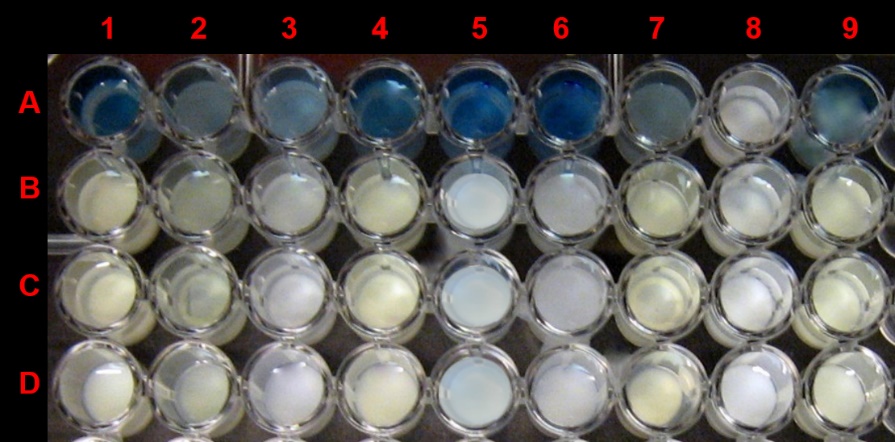


Figure S5 – Colorimetric assays of all synthesized WO_3_ nanoparticles at 5 g/L. The samples were added in the following order: WO_3_ nanoparticles synthesized from Na_2_WO_4_·2H_2_O and NaCl (column 1 – 3), from Na_2_WO_4_·2H_2_O and Na_2_SO_4_ (column 4 – 6) and from PTA precursor (column 7 – 9), at pH 0.0, 0.4 and 1.8, respectively. In this assay *Geobacter sulfurreducens* was used as positive control (line A), *Escherichia coli* and the buffer as negative controls (line B and C, respectively), and a blank test was left as line D.

**3.1 Statistical analysis**

The statistical analysis of the colorimetric assay with 15 g/L *h*-WO_3_ nanoparticles dispersion reveals a clear statistically significant difference between a positive and negative sample (P < 0.0001), proving the specificity of the developed paper-based device (Table S1).

Table S2 - Tukey's Multiple Comparison Test for EAB detection platform.

|  | **Mean Diff.** | **q** | **Significance**  **P < 0.0001** | **Summary** | **99.9 % CI of diff** |
| --- | --- | --- | --- | --- | --- |
| ***Gs* vs *E. coli*** | 0.3408 | 211.9 | Yes | *** | 0.3167 to 0.3649 |
| ***Gs* vs Blank** | 0.3439 | 213.6 | Yes | *** | 0.3200 to 0.3715 |

1. ***Geobacter sulfurreducens* characterization**

The bacterial growth was followed through absorbance readings at 600 nm (Figure S6), to further knowledge of the time required for *Geobacter sulfurreducens* cells reach a certain growth phase.


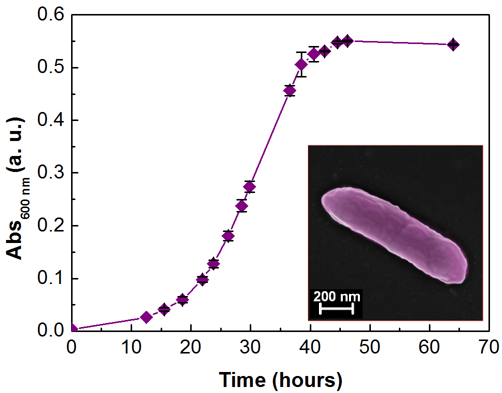


Figure S6 –Growth curve of *Geobacter sulfurreducens*. The inset represents a SEM image of the organism.
